# Accumulation of cytokinins in roots and their export to the shoots of durum wheat plants treated with the protonophore carbonyl cyanide *m*-chlorophenylhydrazone (CCCP)

**DOI:** 10.1093/jxb/eru113

**Published:** 2014-04-01

**Authors:** Guzel R. Kudoyarova, Alla V. Korobova, Guzel R. Akhiyarova, Tatiana N. Arkhipova, Denis Yu. Zaytsev, Els Prinsen, Naum L. Egutkin, Sergey S. Medvedev, Stanislav Yu. Veselov

**Affiliations:** ^1^Institute of Biology, Ufa Research Centre, Russian Academy of Sciences, pr. Oktyabrya 69, 450054, Ufa, Russia; ^2^Department of Biology, Laboratory for Plant Biochemistry and Physiology, University of Antwerpen, 2020 Antwerpen, Belgium; ^3^Institute of Organic Chemistry, Ufa Research Centre, Russian Academy of Sciences, pr. Oktyabrya 69, 450054, Ufa, Russia; ^4^St Petersburg State University, Universitetskaya naberezhnaya 7/9, 199034, St Petersburg, Russia; ^5^Bashkir State University, Zaki-Validi st. 32, 450074, Ufa, Russia

**Keywords:** Cytokinin, protonophore, root-to-shoot communication, secondary active uptake, *Triticum durum*, xylem transport, zeatin.

## Abstract

Cytokinin flow from roots to shoots can serve as a long-distance signal important for root-to-shoot communication. In the past, changes in cytokinin flow from roots to shoots have been mainly attributed to changes in the rate of synthesis or breakdown in the roots. The present research tested the possibility that active uptake of cytokinin by root cells may also influence its export to shoots. To this end, we collapsed the proton gradient across root membranes using the protonophore carbonyl cyanide *m*-chlorophenylhydrazone (CCCP) to inhibit secondary active uptake of exogenous and endogenous cytokinins. We report the impact of CCCP on cytokinin concentrations and delivery in xylem sap and on accumulation in shoots of 7-day-old wheat plants in the presence and absence of exogenous cytokinin applied as zeatin. Zeatin treatment increased the total accumulation of cytokinin in roots and shoots but the effect was smaller for the shoots. Immunohistochemical localization of cytokinins using zeatin-specific antibodies showed an increase in immunostaining of the cells adjacent to xylem in the roots of zeatin-treated plants. Inhibition of secondary active cytokinin uptake by CCCP application decreased cytokinin accumulation in root cells but increased both flow from the roots and accumulation in the shoots. The possible importance of secondary active uptake of cytokinins by root cells for the control of their export to the shoot is discussed.

## Introduction

Cytokinin hormones influence numerous developmental processes in plants. In xylem sap, cytokinin concentrations change in response to external factors such as availability of nitrates (Takei *et al.*, 2002; Rahayu *et al.*, 2005; Vysotskaya *et al.*, 2009), salinity (Ghanem *et al.*, 2011), and water shortage (Kudoyarova *et al.*, 2007; Alvarez *et al.*, 2008). These changes correlate with the cytokinin-controlled processes in the shoots, such as the expression of numerous genes (Brenner and Schmülling, 2012), adjustments in stomatal conductance (Vysotskaya *et al.*, 2010), and the extent of leaf senescence (Dong *et al.*, 2008), suggesting a causal relationship. Since cytokinins are synthesized in roots (Miyawaki *et al.*, 2004), these results suggest the involvement of root-sourced cytokinins in communication between roots and shoots (long-distance signalling). However, some experiments indicate that shoot development is affected more by cytokinin from the shoot itself rather than by supply from the roots (Faiss *et al.*, 1997). In other cases, local increases in cytokinin production by roots have been linked to a delay in leaf senescence (Dong *et al.*, 2008) and stomatal opening (Vysotskaya *et al.*, 2010). The most convincing example implicating root-sourced cytokinins in root–shoot signalling is the effect of changes in nitrate supply to roots (Takei *et al.*, 2002). The balance of evidence favours the view that cytokinin flow from the roots controls a number of developmental processes in shoots.

Changes in the concentration of cytokinins in xylem sap to the shoots have been mostly attributed to changes in cytokinin synthesis in roots (Takei *et al.* 2001; Rahayu *et al.*, 2005) or to their decay catalysed by cytokinin oxidases located close to sites of xylem loading (Brugiere *et al.*, 2003). There is, however, a third potential mechanism controlling cytokinin loading to xylem. This is the transmembrane transport of cytokinins by root cells. Such transport may be mediated by purine permease (*PUP*) and equilibrium nucleoside transport proteins (*ENT*) that are an integral part of lipid membranes. Genes coding for such permeases are known in *Arabidopsis thaliana* and rice (Burkle *et al.*, 2003; Hirose *et al.*, 2005). However, a recent review on cytokinin transport stated that the functional importance for plant growth and development of these putative transmembrane cytokinin transporters remains unproven (Muraro *et al.*, 2011). Nevertheless, there is experimental support of their involvement since increases in expression of *PUP* and *ENT* have been detected in cells adjacent to the vasculature (Aloni *et al.*, 2005). Aloni suggested that, in leaves, these putative cytokinin transporters are involved in both the retrieval of cytokinins unloaded from xylem and their loading to phloem. However, their involvement in xylem loading has not been considered hitherto, although high expression of *ENT2* genes has been observed in roots of *Arabidopsis* plants (Hirose *et al.*, 2005). In root cell suspension cultures, the importance of energizing cytokinin transport has been demonstrated with the help of carbonyl cyanide *m*-chlorophenylhydrazone (CCCP), a protonophore that collapses proton gradients across membranes thereby inhibiting secondary active transmembrane transport (Cedzich *et al.*, 2008). Secondary active uptake is energized by an electrochemical gradient created by proton pumping. In the present experiments, we studied the effects of CCCP on accumulation of cytokinins in root cells, their distribution between roots and shoots, concentration in xylem sap, and rates of delivery to shoots of durum wheat plants in the presence and absence of exogenous zeatin at the roots. Our goal was to discover if active cytokinin transport can influence cytokinin distribution within the root and between root and shoot.

## Materials and methods

### Plant material

Laboratory experiments were performed on 7-day-old seedlings of durum spring wheat (*Triticum durum* Desf., cv. Bezenchukskaya 139). Seeds were germinated in darkness on rafts made from sealed glass tubes tied together and floated on tap water at 24 °С for 3 days and then suspended over 0.1 strength Hoagland–Arnon nutrient medium in 3-litre containers and grown at an irradiance of 400 µmol m^–2^ s^–1^ and a 14-h photoperiod for 6 days. The air temperature was 22–25 °С. Ten 6-day-old seedlings were planted in vessels containing 100 ml of full-strength Hoagland–Arnon nutrient medium for adaptation. Experiments were performed on 7-day-old seedlings. *Trans*-zeatin (Z+) (Sigma, USA) was dissolved in a minimum of ethanol and sufficient amount added to half of the vessels to make a final concentration of 4.0 × 10^–7^ M. To inhibit active cytokinin uptake by cells, the protonophore cyanide *m*-chlorophenylhydrazone (CCCP, Sigma, USA) was added to the nutrient solution at the same time as zeatin to yield 10 µM concentration (CCCP was also added to the controls without zeatin).

After roots had been treated for 1 h, root-xylem exudate was collected as described by Vysotskaya et al. (2009, 2010). For this, the seedlings were cut under water at the root–shoot junction and the shoots reconnected to the roots under water with fine silicon tubes. A comparison of the transpiration rate of intact and cut and rejoined plants by the weighing technique showed that this procedure did not affect transpiration significantly. After 10 min, tubes from 20 plants were disconnected and the root exudate within the tubes weighed and sampled for cytokinin.

### Cytokinin analysis

Concentrations of cytokinin (zeatin, its riboside, nucleotide, *O*-glucoside and *N* ^9^-glucoside) in the roots and shoots were determined 1 h and 6 h after the start of the experiment. Cytokinin purification and enzyme immunoassay were as described by Kudoyarova *et al.* (2007). Shoots and roots were homogenized and cytokinins extracted in 80% ethanol. The extract was separated from plant debris by centrifugation and the ethanol evaporated to leave an aqueous residue. Aliquots of the aqueous residue or of xylem root exudate were loaded on a C18 cartridge (500 mg, Varian, Middelburg, The Netherlands), which was then washed with 20 ml of distilled water. Cytokinins were eluted with 70% ethanol and the eluate evaporated to dryness and dissolved in a minimum of 80% ethanol. This was loaded on precoated 5 × 20 cm, 0.25 mm thick silufol 60 F-254 plates (Merck, Darmstadt, Germany) for thin layer chromatography in the solvent system of butanol, ammonium hydroxide, and water (6:1:2). After ultraviolet detection of standard zeatin, its nucleotide, glucoside and riboside in a separate track for standards, the corresponding zones from the plant material were eluted with 0.1 M phosphate buffer (PB, pH 7.2–7.4). This protocol successfully separated and assayed zeatin nucleotide (Rf 0–0.1), zeatin glucoside (Rf 0.1–0.2), zeatin riboside (Rf 0.4–0.5), and zeatin (Rf 0.6–0.7) (Vysotskaya *et al.*, 2009). More than 90% recovery was obtained for zeatin, its riboside and glucoside standards. Anti-cytokinin antibodies with high immunoreactivity towards *trans*-zeatin, its riboside, *N* ^9^-glucoside and nucleotides showed an inherently low cross-reactivity to dihydrozeatin and isopentenyladenine (iPA) and their derivatives. Since *O*-glucosides have very low affinity to the antibodies used in this work, treatment with β-glucosidase (1 mg enzyme from Sigma, USA per ml sample from 0.1 g of fresh leaves) was carried out for 4 h at 37 °C, pH 5 to release immunoreactive zeatin for glucoside quantification (Arkhipova *et al.*, 2007).

The aforementioned method was validated for our plant material by LC-MS/MS. Prior to LC-MS/MS quantification, cytokinin extraction and purification were undertaken according to Redig *et al.* (1996). Thus samples were homogenized in liquid nitrogen and extracted overnight at –20 °C in Bieleski solution (Bieleski, 1964). Deuterated cytokinins [^2^H_3_]HZ, [^2^H_3_]HZR, [^2^H_5_]HZ-N^9^-G, [^2^H_3_]HZR-P (8 pmol, OlChemIm, Olomouc, Czech Republic) were added as internal standards. After centrifugation (24000 *g*, 4 °C, 15 min), the supernatants were collected and the pellet resuspended for 1 h at 4 °C in 80% MeOH and centrifuged a second time at 14000 rpm for 15 min. Cytokinins were purified by combined solid-phase extraction and immunoaffinity chromatography using anti-cytokinin immunoaffinity columns (OlChemIm, Olomouc, Czech Republic). This purification involved separating the extracted cytokinins into two fractions with reverse-phase C18 cartridges that retained the cytokinin-free bases, ribosides and *N* ^9^-glucosides, respectively. These cytokinins were then further purified using immunoaffinity chromatography. Finally, cytokinin samples were analysed using a ACQUITY UPLC TQD-MS/MS EQUITY (Waters, Micromass Ltd, Milford, MA, USA) following Prinsen *et al.* (1998). Six microlitres (partial loop) were injected onto a BEH C18 column (2.1 × 50 mm, 1.7 µm, Waters) fitted with a VanGuard Pre-Column (2.1 mm, Waters). The mobile phase for elution was 1 mM ammonium acetate (solvent A) and methanol (100%) (solvent B). Quantification was performed by Multiple Reaction Monitoring (MRM) of [MH]^+^ combined with the m/z of the appropriate product ion (Prinsen *et al.*, 1995). The chromatograms obtained were processed using Masslynx 3.4 software (Waters, Milford, MA, USA).

### Cytokinin localization

For cytokinin localization within root tissues (Korobova *et al.*, 2013), sections were cut 1.5 mm from root tips and fixed in 4% paraformaldehyde (Riedel-deHaen, Germany) and 0.1% glutaraldehyde (Sigma, Germany). The tissue samples were then washed with 0.1 M PB (pH 7.2–7.4) for 1 h dehydrated in a series of ethanol dilutions and embedded in hydrophilic methylacrylate resin JB-4 (Electron Microscopy Sciences, USA). Histological sections 1.5 µm thick were cut on a rotary microtome (HM 325, MICROM Laborgerate, Germany). Hormone immunolocalization was performed as described earlier (Veselov *et al.*, 1999; Korobova *et al.*, 2013). Sections were treated with 0.1 M PB (pH 7.2–7.4) containing 0.2% gelatin and 0.05% Tween 20 (PGT) for 30 min. Then, 20 µl of rabbit serum containing antibodies against cytokinins and diluted with PGT (1:80) were placed on the sections. The sections were covered with Parafilm and incubated in the humid chamber at room temperature for 2 h. After incubation, the sections were washed three times (10 min each) with 0.1 M PB containing Tween 20 at the final concentration of 0.05% (PT). Thereafter, 20 µl of goat anti-rabbit immunoglobulins (Aurion, USA) labelled with colloid gold and diluted with PGT (1:40) were placed on each section. Sections were covered with Parafilm and incubated for 1 h in a humid chamber at room temperature before washing three times (10 min each) in PT, postfixing in 2% glutaraldehyde (Sigma, Germany) in PB for 5 min and incubating with silver enhancer (Aurion, USA) for 20 min. Tissue samples were treated with non-immune rabbit serum as control. All preparations were examined with the Axio Imager.A1 light microscope (Carl Zeiss Jena, Germany) equipped with an AxioCam MRc5 digital photocamera (Carl Zeiss Jena). The reliability of the technique for immunohistochemical localization was confirmed by the absence of immunostaining when anti-cytokinin serum was substituted with the non-immune serum.

Determination of octanol–water partition coefficient (*K*_OW_) isotherm for extraction was obtained by mixing of *n*-octanol and solutions of zeatin and zeatin riboside in PB (pH 6.5). Equilibrium concentrations for the partitioned zeatin (1) and its riboside (2) in aqueous (*X*) and organic (*Y*) phases was read as ultraviolet absorbance measurements at 25 °C at 269 and 267 nm, respectively. *K*_OW_ was calculated as *Y*/*X*.

All experiments were repeated three times with three replicates for each treatment.

## Results

**Fig. 1.**
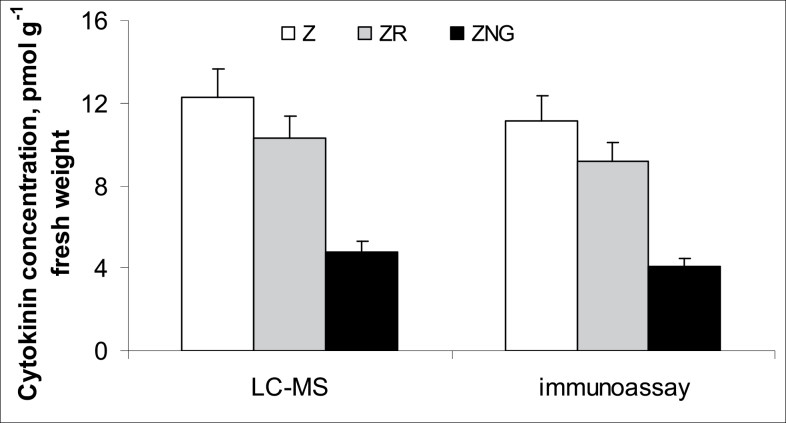
Naturally occurring concentrations of zeatin (Z), zeatin riboside (ZR) and zeatin *N* ^9^-glucoside (ZNG) expressed on a fresh weight basis in untreated whole wheat seedlings at the one-leaf stage (7 days old) measured by LC-MS and immunoassay.

**Fig. 2.**
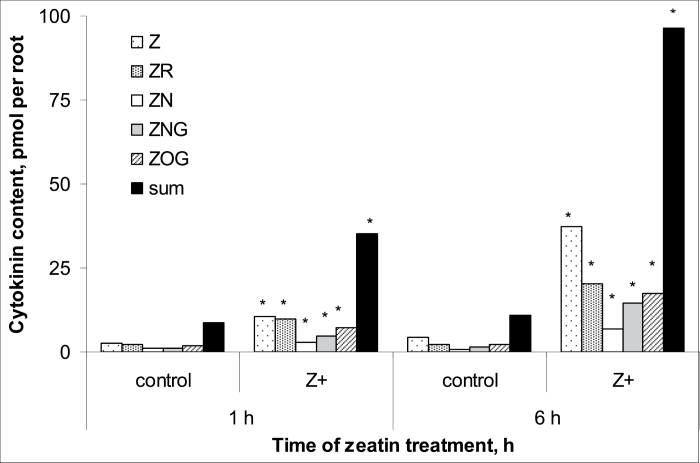
Cytokinin content [zeatin (Z), its riboside (ZR), nucleotide (ZN), *N*-glucoside (ZNG), and *O*-glucoside (ZOG) and their sum] of roots of 7-day-old wheat plants after 1 h or 6 h exposure to exogenous zeatin (Z+, 4 × 10^ –^7 M). Controls were not treated with zeatin. Statistically significant differences (*t*-test, *P* <0.05) between control and treated plants are indicated by an asterisk.

**Fig. 3.**
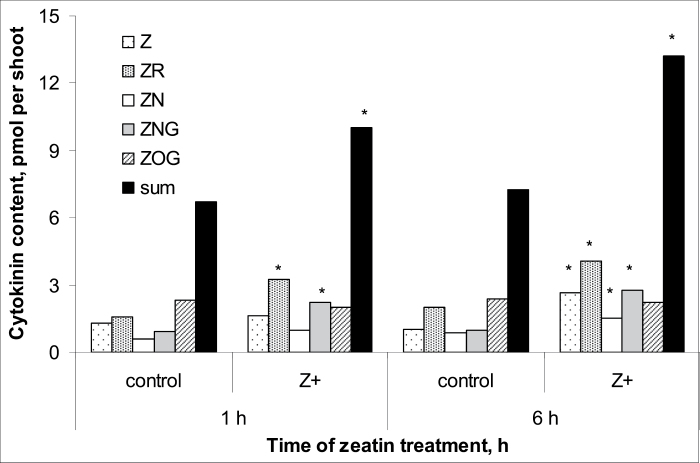
Cytokinin content [zeatin (Z), its riboside (ZR), nucleotide (ZN), *N*-glucoside (ZNG), and *O*-glucoside (ZOG) and their sum] of shoots of 7-day-old wheat plants 1 and 6 h after exposure to exogenous zeatin (Z+, 4 × 10^ –^7 M). Controls were not treated with zeatin. Statistically significant differences (*t*-test, *P* <0.05) between control and treated plants are indicated by an asterisk.

**Fig. 4.**
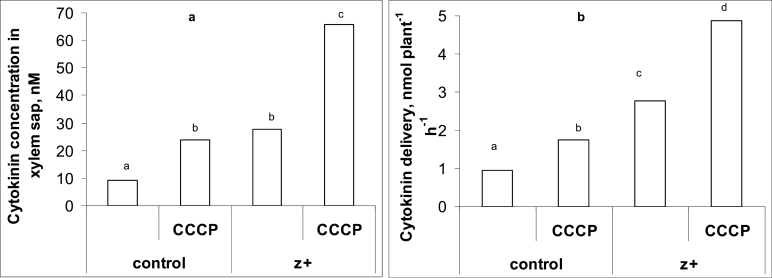
Concentrations (a) and delivery rates from roots to shoots (b) of total cytokinins in xylem sap of 7-day-old wheat plants after 1-h exposure to exogenous zeatin (Z+, 4 × 10^–7^ M). Controls were not treated with zeatin. Statistically different means are indicated by different letters (*P* <0.05, LSD test).

Comparison of the measurements of different derivatives of *trans*-zeatin in wheat plants by means of either immunoassay or physicochemical analyses using LC-MS/MS showed that the methods gave very similar results and means were statistically indistinguishable (Fig. 1). Thus, in subsequent analyses, immunoassays alone were used to quantify cytokinins. The absolute levels of active cytokinins (i.e. zeatin and its riboside) were higher and those of the glucoside lower than those reported previously for wheat by Kosova *et al.* (2012). This may be explained by the difference in plant age. Kosova *et al*. used plants at the three-leaf stage while we studied plants at the one-leaf stage. Treatment of the roots with exogenous zeatin increased total cytokinin content, with much more cytokinin accumulating in the roots compared with the shoots (Figs 2 and 3). One hour after the start of the treatment, cytokinin in roots increased 4-fold, while content in the shoot was only 1.5 times greater than in controls. Accumulation of cytokinin in roots continued with time, and 6 h after the start of exposure to exogenous zeatin total content exceeded the control level 9-fold. By this time, shoot cytokinin level was only 1.8 times more than in controls. Concentration of cytokinins in xylem sap was also increased by the treatment (Fig. 4a). When the flux of cytokinin from roots to shoots (the delivery rate) was calculated by multiplying concentration by the rate of transpiration, this was found to be much increased by zeatin treatment (Fig. 4b). This was expected since transpiration of the wheat plants was not changed by zeatin treatment; control and zeatin-treated plants each lost about 100 mg of water per hour. Although plants were treated with the free base, it was not only the content of this form of cytokinin that accumulated in the plants. Increases were also seen in zeatin riboside, zeatin *N*-glucoside, and zeatin nucleotide. This agrees well with the ready interconversion of free bases, their ribosides and nucleotides reported by Mok and Mok (2001) and the well-established inactivation of cytokinins effected through the irreversible conversion of zeatin into *N*-glucosides (Stirk *et al.*, 2012). The content of zeatin *O*-glucoside was increased by zeatin treatment only in roots, but not in shoots (Figs 2 and 3). This is likely to mean that zeatin *O*-glucoside is not released from xylem parenchyma cells to xylem vessels.

**Fig. 5.**
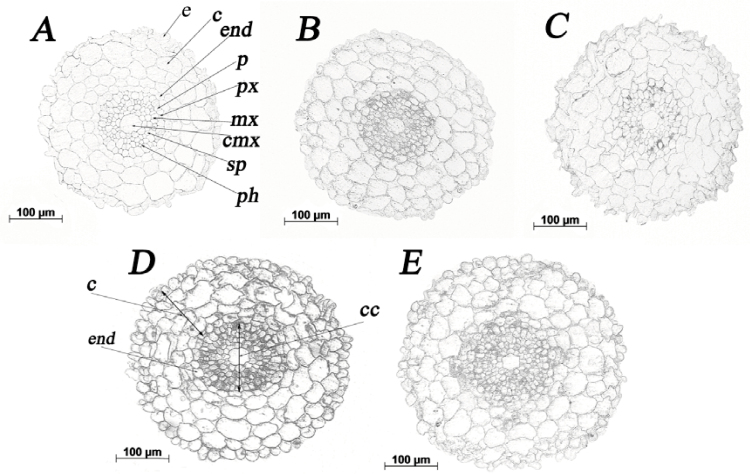
Immunolocalization of zeatin in radial sections of wheat roots after 1 h exposure to exogenous zeatin (Z+, 4 × 10^–7^ M). Sections were taken 1.5 mm behind the tip: (A) root incubated with non-immune serum; (B) control root; (C) control root treated with 10 µM carbonyl cyanide *m*-chlorophenylhydrazone (CCCP); (D) zeatin-treated root; (E) roots treated with zeatin and CCCP. e, epidermis; c, cortex; end, endodermis; p, pericycle; px, protoxylem; mx, metaxylem; cmx, central metaxylem; sp, stele parenchyma; ph, phloem.

Immunolocalization of zeatin in root sections cut 1.5 mm from its tip was carried out using antibodies to zeatin riboside. This allowed zeatin to be localized because (i) the fixation process enabled zeatin to conjugate to adjacent protein while losing the riboside and (ii) the zeatin riboside antibody readily recognizes zeatin (Veselov *et al.*, 1999). The results showed an uneven distribution of zeatin between different parts of the root. Cells inside the endodermis were more intensively immunostained than those of the cortex (Fig. 5B). Unlike root sections of control plants, immunolabelling for cytokinins in zeatin-treated plants was also detected in some cells of the exodermis (Fig. 5D). As expected, exposure of the roots to zeatin increased the intensity of immunostaining. This served as confirmation of the specificity of the technique for localizing zeatin.

**Fig. 6.**
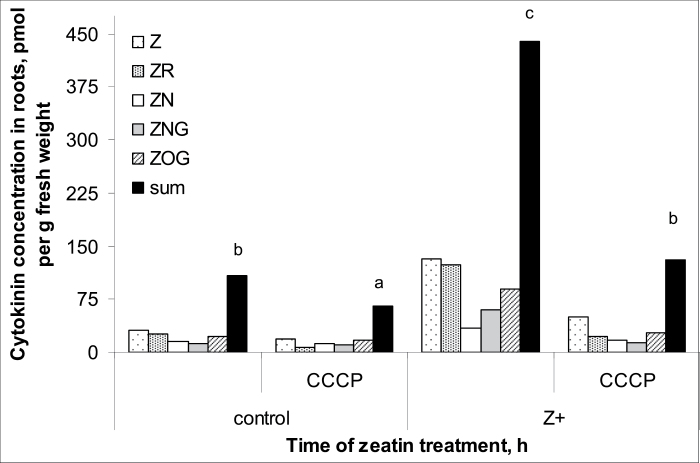
Cytokinin concentration [zeatin (Z), its riboside (ZR), nucleotide (ZN), *N*-glucoside (ZNG), and *O*-glucoside (ZOG) and their sum] in roots of 7-day-old wheat plants after 1-h exposure to exogenous zeatin (Z+, 4 × 10^–7^ M) and 10 µM carbonyl cyanide *m*-chlorophenylhydrazone (CCCP). Statistically different means are indicated by different letters (LSD, *P* <0.05).

The importance of secondary active cytokinin uptake (i.e. utilizing the energy derived from the transport of H^+^ down its concentration gradient) for accumulation of cytokinins of root cells was first suggested by experiments with the protonophore CCCP in cell suspension cultures (Burkle *et al.*, 2003; Cedzich *et al.*, 2008). Addition of CCCP was therefore used in the present experiments to examine the possibility that secondary active cytokinin uptake by the cells can affect the distribution of cytokinins between root cells and between the roots and shoots via transport in the xylem sap. One hour of CCCP treatment was chosen to minimize any indirect effects that may result from longer-term action of the protonophore. Application of CCCP decreased total root cytokinin content in both the control and zeatin-treated plants (Fig. 6). All five cytokinins assayed (zeatin, zeatin riboside, zeatin nucleotide, zeatin *N*-glucoside and zeatin *O*-glucoside) were decreased. Comparison of the level of immunostaining in the root sections treated with CCCP also showed a decline in intensity of immunolabelling resulting from the inhibition of secondary active cytokinin uptake by CCCP (Fig. 5C, E).

**Fig. 7.**
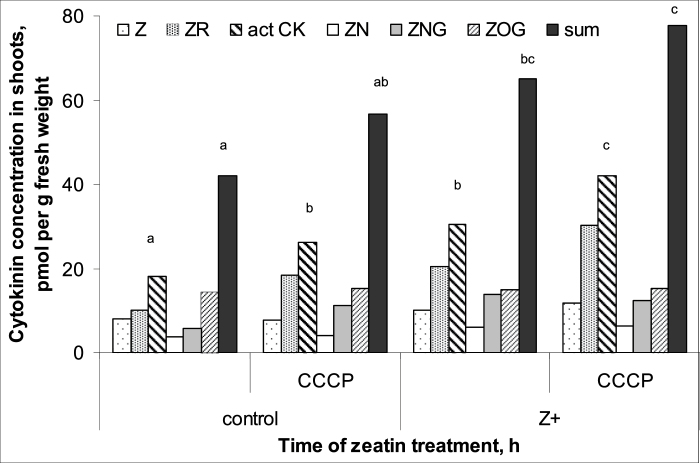
Cytokinin concentration [zeatin (Z), its riboside (ZR), active cytokinins (act cyt: Z+ZR), nucleotide (ZN), *N*-glucoside (ZNG), and *O*-glucoside (ZOG) and their sum] in shoots of 7-day-old wheat plants after 1-h exposure to exogenous zeatin (Z+, 4 × 10^–7^ M) and 10 µM carbonyl cyanide *m*-chlorophenylhydrazone (CCCP). Statistically different means are indicated by different letters (LSD, *P* <0.05).

In contrast to the roots, CCCP treatment increased the concentration of active cytokinins (zeatin and its riboside) in shoots of both control and zeatin-treated plants (Fig. 7). Total concentration of cytokinins appeared to be increased but not to a statistically significant extent. This arose because CCCP failed to change the concentration of *O*-glucosides. Analyses of xylem sap (Fig. 4a) revealed that CCCP treatment increased the concentration of total cytokinins in xylem sap considerably. To determine whether more cytokinin was being delivered from roots to shoots in this sap, concentrations were multiplied by the rate of sap flow (i.e. the rate of transpiration) to calculate delivery. Transpiration rate was not the same in all plants since CCCP treatment reduced it by approximately 25%. The calculations (Fig. 4b) show that the delivery of cytokinins from roots to the shoot was increased in both control and zeatin-treated plants by about 80% when CCCP was applied.

**Fig. 8.**
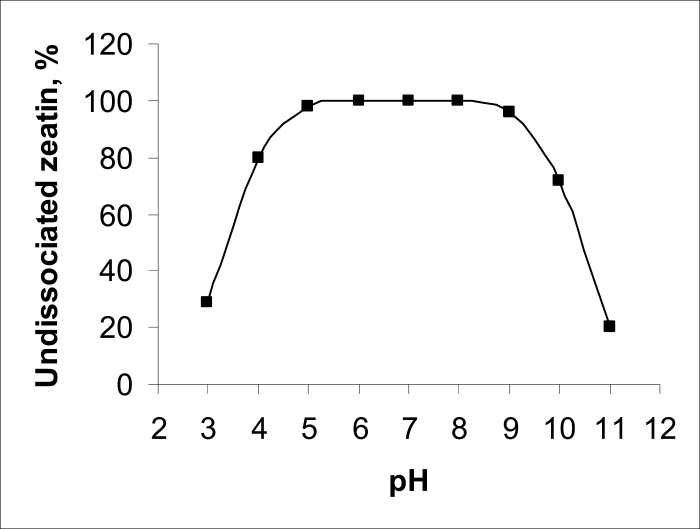
Effect of increasing pH on the percentage of undissociated molecules of zeatin in aqueous solution calculated according to equation: percent of undissociated molecules = 100/(1 + *K*_a_/[H^+^] + *K*_b_[H^+^])/*K*_w_, where *K*_a_ and *K*_b_ are the acidic and basic dissociation constants of zeatin, p*K*_a_=10.4 and p*K*_b_=3.6 (Yokota *et al.*, 1980), and *K*_w_ is dissociation constant of water (p*K*_w_=1 × 10^–14^).

All the zeatin is in an undissociated molecular form in the physiological range of pH between 5 and 9 (Fig. 8).

## Discussion

Zeatin and its riboside are membrane-permeable cytokinins (Glover *et al.*, 2008) and some early publications report passive uptake of the free base of the synthetic cytokinin benzyladenine (Lampugnani *et al.*, 1981). Passive cytokinin transport through xylem regulated by the transpiration stream has been hypothesized (Ramina *et al.*, 1979). Partition coefficients between octanol and aqueous phases (pH 6.5) determined at 25 °C were 2.9 and 0.55 for zeatin and its riboside, respectively, showing that the lipophilicity of zeatin is about five times greater than that of its riboside due to hydration of sugar moieties. So it is possible that the increase in zeatin content in roots of plants incubated on solution of zeatin could be due merely to the passive movement of this hormone down an inwardly directed concentration gradient (about 3 × 10^–8^ M in the cells and 4 × 10^–7^ M of zeatin in the medium). However, passive zeatin transport cannot explain how cytokinins are retained in root cells. A study of auxin transport has shown that because of the neutral pH of cytoplasm, the majority of auxin molecules exist in the dissociated form and, as a result, are unable to pass through the plasma membrane by simple diffusion. As a result, indole acetic acid becomes trapped within cells and can leave them only by the action of efflux carriers (Tanaka *et al.*, 2006; Medvedev, 2012). However, dissociation trapping is unlikely for zeatin, since it is in an undissociated molecular form in the physiological range of pH between 5 and 9 and thus is able to diffuse readily through membranes (Fig. 8). Therefore the retention of cytokinins inside root cells needs an alternative explanation.

Energized transport of zeatin has been demonstrated in suspension cultures of *Arabidopsis* root cells (Cedzich *et al.*, 2008). This was attributed to expression in the roots of *PUP* genes coding for putative transporters of cytokinin bases (Burkle *et al.*, 2003). Although cytokinins were supplied to the wheat plants in the form of free base, they were converted into zeatin riboside inside the roots. The cell membrane is likely to be less permeable for zeatin riboside than for its free base, although zeatin riboside is still capable of diffusing passively out of cells. Consequently, retention of cytokinins in root cells may be due to activity of not only *PUP* but also *ENT* transporters for cytokinin ribosides, enabling their energized uptake by cells against a concentration gradient (Hirose *et al.*, 2005).

Expression of the gene coding for the AtPUP2 transporter has been found in shoot parenchyma cells around the vasculature, suggesting its involvement in retrieving cytokinins from the symplast to the apoplast for transport to the shoot through xylem (Aloni *et al.*, 2005). In our experiments, accumulation of cytokinins in parenchyma cells around xylem vessels in the root central cylinder was reduced by the treatment of wheat plants with the protonophore CCCP. Since CCCP inhibits cytokinin uptake into xylem parenchyma cells, the cytokinin concentration in the apoplast should be increased and these cytokinins could be loaded easily into the xylem vessels, because mature xylem vessels are part of the apoplast. It must be borne in mind that free bases such as zeatin have to be conjugated to adjacent proteins and their subsequent immunolocalization will reflect not only the distribution of zeatin but also of the proteins to which they bind. However, the decline in immunolabelling resulting from CCCP treatment confirms that the more intensive staining of the cells of the central cylinder reflect cytokinin accumulation rather than higher concentrations of proteins in these cells.

This implies that levels of symplastic zeatin were maintained by the energized uptake of cytokinins by cells. Thus, for zeatin to become loaded into water-conducting xylem vessels, the ratio of cytokinin efflux and influx in and out of adjacent parenchyma cells must shift in favour of efflux. In the present work, this was achieved by inhibiting active uptake of cytokinin with CCCP through the dissipation of the inwardly directed electrochemical gradient in cytokinin. In this way CCCP treatment is thought to increase the release of zeatin into the xylem across the plasma membranes of the xylem parenchyma cells.

When we examined the amounts of zeatin in the transpiration stream, CCCP treatment increased the concentration of both endogenous and exogenously supplied cytokinin. To ensure this indicated a true increase in the flux (delivery) of cytokinin to the shoots from the roots, we calculated this in terms of nanomoles transported per plant per hour after correcting for the slowing effect of CCCP on transpiration. The outcome was an ~80% increase in delivery. The transport of this additional cytokinin out of root cells is probably facilitated by ABC transporters known to participate directly in the transport of a wide range of molecules across cell membranes (Wanke and Kolukisaoglu, 2010).

Less dense immunostaining of the cortex (including cells of endodermis) compared with the cells of the central cylinder suggests that zeatin was transported from the external medium and through the cortex by an apoplastic pathway and that its active uptake occurred in the cells of the central cylinder. The results also indicate that zeatin may be transported by solvent drag across the Casparian bands of the endodermis and that the endodermis is therefore unlikely to be an effective barrier for zeatin in wheat plants of this age.

Although the importance of cytokinins as long-distance signals has sometimes been doubted (Faiss *et al.*, 1997), it has been supported by many other experiments (e.g. Takei *et al.*, 2002; Dong *et al.*, 2008; Vysotskaya *et al.*, 2010). Changes in cytokinin flow from the roots resulting from external influences are likely to be important for the control of adaptive responses (Jackson, 1993; Alvarez *et al.*, 2008; Hirose *et al.*, 2008). They were mostly attributed to changes in cytokinin synthesis or to rates of decay in the roots (Takei *et al.*, 2002; Brugiere *et al.*, 2003; Rahayu *et al.*, 2005). Our results show that changes in active cytokinin uptake by the root cells may, indirectly, influence cytokinin export from the roots. Thus, cytokinin increase in xylem sap and shoots can result from inhibition of their accumulation in root cells. Cytokinins retained in root cells may serve as a reserve that is readily re-mobilized on demand. In our experiments, plants were treated with *trans*-zeatin and antibodies raised against this zeatin isomer had low immunoreactivity to *cis*-zeatin (Arkhipova *et al.*, 2005). High concentration of *cis*-zeatin as well as *trans*-zeatin derivatives have been detected in wheat plants (Kosova *et al.*, 2012). It remains to be discovered in further experiments if secondary active uptake of *cis*-zeatin by root cells is important for the control of their export to the shoot.

Histidine kinase cytokinin receptors were previously believed to bind cytokinins only outside the cells (Haberer and Kieber, 2002). If this is so, localization of cytokinins inside the cells would separate them from their receptors, thereby preventing them from acting physiologically. This view was changed by the discovery of cytokinin receptors on endoplasmic reticulum by Caesar *et al.* (2011), Lomin *et al.* (2011), and Wulfetange *et al.* (2011), showing that cytokinins may be active inside the root cells. This conclusion is also supported by the identification of cytokinin-binding proteins inside root cells (Brovko *et al.*, 2007).

It remains to be established whether changes in zeatin distribution between roots and shoots are important for the control of cytokinin-controlled processes only in shoots or also in roots. The principal finding of the present study is that the regulation of the rate of flow of cytokinin from roots to shoots can be influenced markedly by changes in the uptake and retention of cytokinin by root cells.

## Abbreviations:

CCCP: carbonyl cyanide *m*-chlorophenylhydrazone
PB: phosphate buffer.
